# Role of apparent diffusion map in the evaluation of retinoblastoma

**DOI:** 10.5935/0004-2749.2021-0435

**Published:** 2024-02-10

**Authors:** Viviane Souto Spadoni, Thaylla Maybe Bedinot da Conceição, Flávia da Costa Schaefer, Daniel Schmidt Ercolani, Marcelo Krieger Maestri, Amalia Klaes, Simone Geiger Selistre, Fabiano Reis, Juliana Ávila Duarte

**Affiliations:** 1 Ophthalmology Division, Hospital de Clínicas de Porto Alegre, Porto Alegre, RS, Brazil; 2 Radiology Division, Hospital de Clínicas de Porto Alegre, Porto Alegre, RS, Brazil; 3 Pediatric Oncology, Hospital de Clínicas de Porto Alegre, Porto Alegre, RS, Brazil; 4 Department of Anesthesiology, Oncology and Radiology, Faculdade de Ciências Médicas, Universidade Estadual de Campinas, Campinas, SP, Brazil; 5 Department of Internal Medicine, Faculdade de Ciências Médicas, Universidade Federal do Rio Grande do Sul, Porto Alegre, RS, Brazil

**Keywords:** Retinoblastoma, Prognosis, Retinal neoplasms, Orbit, Diffusion magnetic resonance imaging

## Abstract

**Purpose:**

This study aimed to analyze the association between magnetic resonance imaging apparent diffusion coefficient map value and histopathological differentiation in patients who underwent eye enucleation due to retinoblastomas.

**Methods:**

An observational chart review study of patients with retinoblastoma that had histopathology of the lesion and orbit magnetic resonance imaging with apparent diffusion coefficient analysis at *Hospital de Clínicas de Porto Alegre* between November 2013 and November 2016 was performed. The histopathology was reviewed after enucleation. To analyze the difference in apparent diffusion coefficient values between the two major histopathological prognostic groups, Student’s t-test was used for the two groups. All statistical analyses were performed using SPSS version 19.0 for Microsoft Windows (SPSS, Inc., Chicago, IL, USA). Our institutional review board approved this retrospective study without obtaining informed consent.

**Results:**

Thirteen children were evaluated, and only eight underwent eye enucleation and were included in the analysis. The others were treated with photocoagulation, embolization, radiotherapy, and chemotherapy and were excluded due to the lack of histopathological results. When compared with histopathology, magnetic resonance imaging demonstrated 100% accuracy in retinoblastoma diagnosis. Optic nerve invasion detection on magnetic resonance imaging showed a 66.6% sensitivity and 80.0% specificity. Positive and negative predictive values were 66.6% and 80.0%, respectively, with an accuracy of 75%. In addition, the mean apparent diffusion coefficient of the eight eyes was 0.615 × 10^3^ mm^2^/s. The mean apparent diffusion coefficient value of poorly or undifferentiated retinoblastoma and differentiated tumors were 0.520 × 10^3^ mm^2^/s and 0.774 × 10^3^ mm^2^/s, respectively.

**Conclusion:**

This study revealed that magnetic resonance imaging is useful in the diagnosis of retinoblastoma and detection of optic nerve infiltration, with a sensitivity of 66.6% and specificity of 80%. Our results also showed lower apparent diffusion coefficient values in poorly differentiated retinoblastomas with a mean of 0.520 × 10^3^ mm^2^/s, whereas in well and moderately differentiated, the mean was 0.774 × 10^3^ mm^2^/s.

## INTRODUCTION

Retinoblastoma is the most common malignancy in children and is more frequently found in children aged <2 years. Most retinoblastomas are unilateral and caused by a spontaneous mutation^(1)^. Conversely, bilateral and multifocal unilateral tumors are heritable in a phenotypically autosomal dominant manner caused by a mutation of the retinoblastoma gene (RB) of chromosome 13q14^(2)^.

Historically, histopathology and clinical parameters of retinoblastoma are related to its prognosis^(3)^. For example, poor histologic differentiation, large tumor size, bilaterality, choroid invasion, and extension post--laminar in the optic nerve have been associated with poor outcomes^(3)^. Moreover, the distinction between poorly and well-differentiated tumors is also important when choosing the best treatment because well-differentiated tumors are radioresistant and poorly differentiated tumors respond to radiation therapy^(4)^.

Although postoperative anatomopathological analysis is the gold standard for the differentiation of retinoblastoma grade, emerging noninvasive studies may provide physiological information related to tissue cellularity. For instance, magnetic resonance imaging (MRI), which is an important option in tumor diagnosis and staging^(5)^, may also detect local extension and metastases^(6)^. In addition, MRI combined with diffusion--weighted imaging (DWI), which is a well-known tool for prognostic evaluation in several malignancies such as breast, prostate, and orbit cancer, may be useful in retinoblastoma^(7,8)^. The quantitative analysis of DWI, as the apparent diffusion coefficient map (ADC), gives values in mm^2^/s for signal strength and may differentiate benign from malignant lesions. Lower ADC map values are attributed to high cellularity and higher nucleus/cytoplasm ratio in poorly differentiated and worse prognosis tumors^(4,7,9,10)^.

In this study, we aimed to examine the association between MRI ADC map values and histopathological differentiation in patients who underwent eye enucleation because of retinoblastomas.

## METHODS

The study included all patients with retinoblastoma who were referred to our hospital between November 2013 and June 2016. All patients underwent funduscopy under general anesthesia before MRI.

The **inclusion criteria** were as follows: (1) availability of diagnostic-quality preoperative contrast-enhanced MRI at 1.5 T, with diffusion-weighted MRI, and (2) histopathologically proven retinoblastoma after enucleation. Histopathology was divided into undifferentiated and differentiated (including well and moderately differentiated tumors).

The **exclusion criteria** were as follows: (1) patients evaluated before and after the study period, (2) patients who underwent embolization, photocoagulation, or radiotherapy and chemotherapy without enucleation, (3) MRI without ADC map evaluation, and (4) poor quality MRI.

Patients’ clinical data including sex, age (years old), laterality (unilateral/bilateral), eye involved (right/left), lesion size (cm), MRI results, treatment method, and final histological outcomes were collected.

The patients underwent MRI with surface coils asso-ciated with an 8-channel skull coil in a 1.5 T Philips before treatment that included the following sequences:

- T2WI = axial and coronal with fat suppression and 4000-4600/19-96 ms of repetition time/echo time (TR/TE), 20 × 22 cm field of view (FOV), 3 mm of thickness, gap of 1 mm, and matrix of 320 × 260.
- DWI = multisection spin-echo echo-planar imaging sequence with 3200/80 ms of TR/TE, 20 × 22 cm of FOV, 3 mm of thickness, gap of 1 mm, excitation number of 6, matrix of 128, EPI factor of 128, radiofrequency pulse of 1200 Hz/pixel, B factor of 0, and 1000 mm^2^/s, and ADC map was mathematically generated.
- T1WI = pre-contrasted with and without fat suppression and T1 post-contrasted fat suppression (TR/TE of 400-575/13-15 ms) sequences in axial, coronal, and sagittal with intravenous injection of 0.1 mL/kg gadopentetate dimeglumine.
- T2 balance = T2-weighted volumetric sequence with 0.5 mm of thickness, 6.7 ms of GRETR, and 3.3 ms of TE.

**Figure 1:**
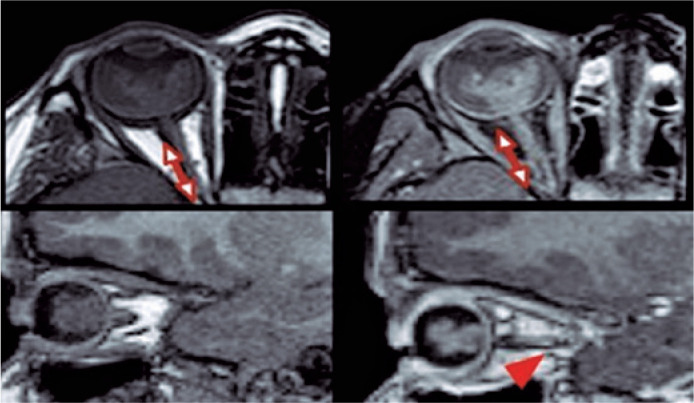
Sagittal and axial T2WI spin-echo images demonstrating a large retinoblastoma in the left eye with a very low signal intensity extending to the anterior chamber.

The lesion size was evaluated on the longer axis at T1WI post-contrast (gadolinium) sequences. On MRI, retinoblastoma was considered when the lesion had moderately higher signal intensity on T1WI and lower signal intensity on T2WI in comparison with the vitreous body. Other imaging signs included globe deformation, increased globe size, and reduced anterior chamber and buphtalmia (Figure 1).

Laterality (right, left, or both eyes), growth pattern (endophytic, exophytic, or mixed), and tumor localization were analyzed. Tumor localization was identified as anterior or posterior to the equator, which is the greatest globe diameter. Moreover, posterior to the equator can be divided into macular, juxtapapillar, and none of the above. Tumor size was measured as its maximum diameter.

MRI parameters for tumor characteristics and invasion of the optic nerve, choroid, sclera, and ciliary body were examined. The maximal tumor diameter on transverse post-contrast T1 was calculated and classified as <10 mm, 10-15 mm, and >15 mm. The presence of tumor calcification was defined by dark signal spots on both T1WI and T2WI without enhancement. For quantitative analysis of diffusion-weighted MRI (DWI), the solid components of the retinoblastoma were identified on T2WI and post-contrast T1WI. Regions of interest (ROIs) were manually placed within the solid part of the tumor to avoid bias from necrotic and hemorrhagic elements, and the ADC map values of the solid components of each tumor were measured. The average ADC map values of the three measurements were used for further analysis. In bilateral tumors, the ADC map value of the enucleated eye was used for further analysis.

Diffusion coefficients were measured on the ADC map, consisting of three measurements, with ROI ranging from 0.5 to 0.3 cm, covering the largest possible area in each lesion and averaging between values.

**Figure 2:**
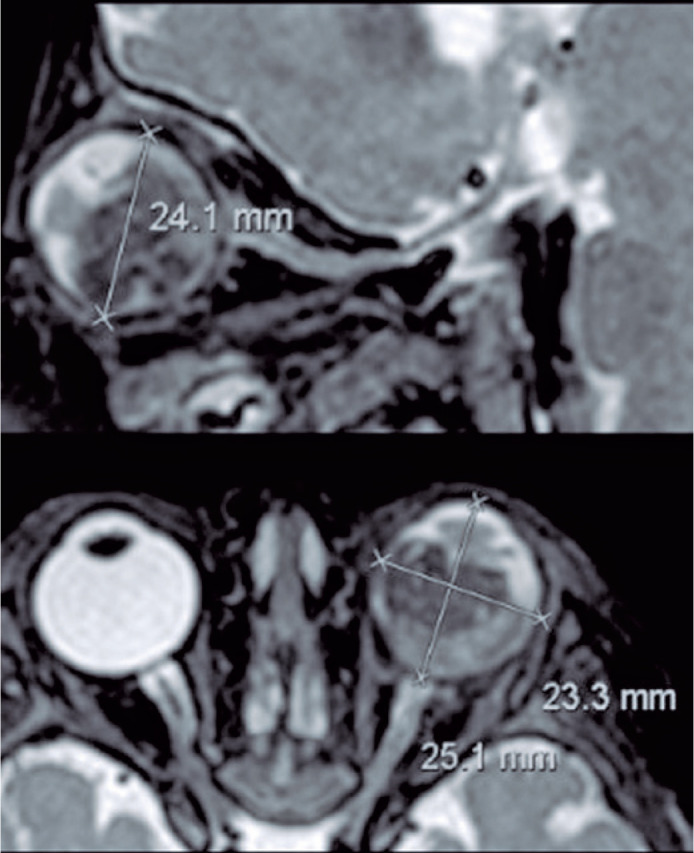
Large retinoblastoma with signs of optic nerve infiltration along its intraorbital segment. Axial abs saggital preand post-gadolinium injection demonstrated strong enhancement of the optic nerve (left column).

Optic nerve involvement was considered when there was thickening, irregular contour, and abnormal enhancement of the optical nerve or discontinuity of the enhancement of the linear choroid-retinal complex. The optic nerve was divided into prelaminar, laminar, and post-laminar segments by the cribriform plate. MRI findings of post-laminar optic nerve infiltration include focal optic nerve enhancement (Figure 2).

The degree of agreement between the findings of the 1.5-T protocol associated with surface coils was analyzed by comparing fundus examination findings and histopathological findings.

Sensitivity, specificity, positive predictive value (PPV), negative predictive value (NPV), and accuracy were estimated for conventional MRI in the detection of the tumor and its extent. All numerical values were expressed as means. First tested using the Kolmogorov-Smirnov test for normality analysis. The intraclass correlation coefficient was used to evaluate the interobserver agreements for ADC values. To examine whether ADC values can provide prognostic information, the difference in ADC values for each prognostic group (tumor size, bilaterality, growth pattern, degree of differentiation, optic nerve invasion, choroidal invasion, scleral invasion, and ciliary body invasion) was analyzed using Students’ t-test for two groups. The receiver operating characteristic curve was used to analyze the diagnostic performance of ADC for predicting risk factors. A p-value <0.05 was considered to establish statistically significant differences. All statistical analyses were performed with IBM SPSS for Microsoft Windows version 19.0 (SPSS, Inc., Chicago, IL, USA).

To analyze the difference in ADC values between the two major histopathological prognostic groups (undiffe-rentiated versus differentiated), Student’s t-test was used for two groups. A p-value of 0.05 was considered to establish statistical significance. All statistical analyses were performed using IBM SPSS for Microsoft Windows version 19.0. Our institutional review board approved this retrospective study and waived the need for informed consent.

## RESULTS

In this study, we evaluated 13 children, 7 boys and 6 girls. Among all patients, 8 underwent eye enucleation and were included in our analysis. The others were treated with photocoagulation, embolization, radiotherapy, and chemotherapy and were excluded because they lack histopathological results (Table 1).

**Table 1:** Radiological and histopathologic features of eight eyes primarily enucleated for retinoblastoma

Patients	MRI optic nerve (on) invasion	Histopathological findings optic nerve (on) invasion	Histopahological grade
Patients 01	Absent	ON invasion	Poorly differentiated
Patients 02	on invasion	ON invasion	Poorly differentiated
Patients 04	Absent	Absent	Poorly differentiated
Patients 07	Cho and ON invasion	ON invasion	Well differentiated
Patients 08	Cho and ON invasion	Absent	Moderately differentiated
Patients 09	Absent	Absent	Poorly differentiated
Patients 11	Absent	Absent	Moderately differentiated
Patients 12	Absent	Absent	Poorly differentiated

* Cho= coroidal invasion.

ON= optic nerve invasion.

**Figure 3:**
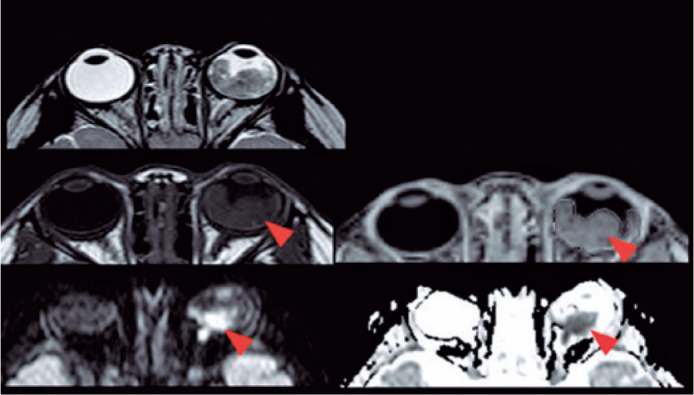
Axial T2WI balance image, axial T2WI spin echo, axial diffusion, ADC map images, T1WI preand post-gadolinium images. Bilateral retinoblastomas have with very low signal intensity on T2WI, restriction diffusion on ADC maps, and gadolinium enhancement on post-contrast images.

MRI demonstrated a 100% accuracy in retinoblastoma diagnosis. Moreover, some subcentimeter lesions (<0.5 cm) were found only in the sequence balance in our protocol (Figure 3).

Optic nerve invasion detection on MRI showed a 66.6% sensitivity and 80.0% specificity when compared with histopathology (gold standard). The PPV was also 66.6%, and the NPV was 80.0% with an accuracy of 75% (Table 2).

**Table 2:** Magnetic resonance imaging and histopathology findings of optic nerve invasion

	MRI with optic nerve (ON) invasion	MRI with optic nerve (ON) invasion	Total
Histopathological with on invasion	2	1	3
Histopathological with on invasion	1	4	5
Total	3	5	8

In addition, the ADC values of the 8 eyes were evalua-ted, and the mean was 0.615 × 10^3^ mm^2^/s. The mean ADC value of poorly or undifferentiated retinoblastoma was 0.520 × 10^3^ mm^2^/s, whereas in differentiated tumors, the mean was 0.774 × 10^3^ mm^2^/s (Table 3).

**Table 3:** ON invasion × histopathological grade × ADC (mean values and the three separate values of each patient)

Patient MRI	MRI optic (ON) invasion	Histopathological findings optic nerve (ON) invasion	Hisyopahological grade	ADC (mean value)	ADC 1	ADC 2	ADC 3
Patients 01	Absert	on invasion	Poorly differentiated	0.54866	0.539	0.61	0.497
Patients 02	on invasion	on invasion	Poorly differentiated	0.32366	0.216	0.419	0.336
Patients 04	Absert	Absert	Poorly differentiated	0.56333	0.55	0.568	0.572
Patients 07	Cho and ON invasion	on invasion	Well differentiated	0.79633	0.623	0.886	0.88
Patients 08	Cho and ON invasion	Absert	Moderately differentiated	0.54233	0.548	0.558	0.521
Patients 09	Absert	Absert	Poorly differentiated	0.51166	0.503	0.558	0.474
Patients 11	Absert	Absert	Moderately differentiated	0.98333	1.003	0.944	1.003
Patients 12	Absert	Absert	Poorly differentiated	0.654	0.624	0.697	0.641

ADC= apparent diffusion coefficient.

## DISCUSSION

Retinoblastoma is a rare eye neoplasia that occurs only during childhood^(2)^. Although histopathological analysis has demonstrated optic nerve invasion and the degree of differentiation is classically associated with high-risk tumors, noninvasive methods that analyze these features before the surgery are controversial^(2)^. For instance, imaging has been demonstrated as an interesting tool to predict these findings. In this scenario, MRI with DWI has been used in the preoperative evaluation to predict histopathological tumor differentiation based on ADC map values^(11)^. We found that MRI is useful in the diagnosis of retinoblastoma and detection of infiltration of the optic nerve with a sensitivity of 66.6% and specificity of 80%.

Previous studies have demonstrated that MRI might predict the degree of optic nerve involvement of retinoblastoma, particularly when considering post-laminar optic nerve invasion^(11,12)^. Cui et al. described in their retrospective study a sensitivity of 73.3% for post-laminar optic nerve invasion. However, their results failed to predict prelaminar (42.9%) and laminar optic nerve invasion for laminar invasion (50.0%).

**Figure 4:**
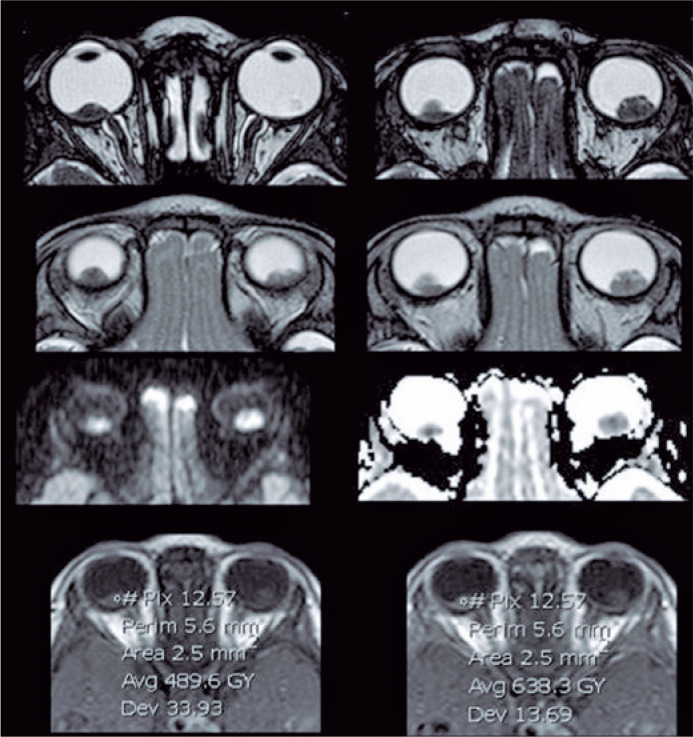
Axial submilimetric T2WI GRE image (balance sequence), axial T2WI spin echo, and axial ADC map showing very low signal intensities and diffusion restriction (ADC map value of 489.6).

In accordance with previous studies, our results also showed lower ADC values in poorly differentiated retinoblastomas with a mean of 0.520 ×10^3^ mm^2^/s, whereas in well and moderately differentiated, the mean was 0.774 × 10^3^ mm^2^/s. Cui et al. evaluated the ADC values of 53 eyes with retinoblastoma and observed lower ADC values in poorly or undifferentiated retinoblastoma (0.74 ± 0.13 × 10^3^ mm^2^/s^^2^^) than those of well-differentiated (0.91 ± 0.14 × 10^3^ mm^2^/s) (p<0.002)^(13)^ (Figure 4).

Razek et al. also found that the mean ADC value was significantly different between well-differentiated (0.54 ± 0.20 × 10^3^ mm^2^/s) and moderately differentiated retinoblastomas (0.51 ± 0.07 × 10^3^ mm^2^/s) compared with poorly differentiated (0.44 ± 0.07 × 10^3^ mm^2^/s) and undifferentiated retinoblastomas (0.41 ± 0.01 × 10^-3^ mm^2^/s)^(4)^.

In accordance with these findings, in orbital tumors, Razek et al. evaluated the difference between malignant and benign lesions at 3-T diffusion MRI in 47 patients and found that malignant lesions had significantly lower mean ADC value (0.84 +/- 0.34 × 10^3^ mm^2^/s) (p=0.001) than benign orbital tumors (1.57 +/- 0.33 × 10^3^ mm^2^/s)^(8)^.

This study has limitations because of its retrospective design and small sample size. Despite these limitations, our results are concordant with published literature. A larger and more comprehensive multicenter study is needed to better describe these findings.

Our ADC values are higher than reported previously; however, evidence shows that the ADC may vary across field strengths and vendors^(14)^. In addition, this difference could be due to our inclusion of moderated and poorly differentiated tumors that had >20% of necrosis, and necrosis is well known to increase the values.

Retinoblastoma is the most common intraocular malignant tumor in childhood, and many options are available for preserving the eye to improve patients’ quality of life, survival, and hope for their families. Thus, more patients are being treated without eye enucleation without histopathological confirmation, making high--resolution orbit MRI with ADC map analysis a very useful tool for tumor grading, local staging, diagnosis, follow-up, recurrence, and evaluation of metastatic disease in these patients. Therefore, radiologists must be familiar with its characteristics and recognize the extent of the tumor and changes that can occur after treatment. When applying high-resolution orbit MRI and ADC map evaluation, more studies are needed to standardize the method and broaden its clinical use.
